# Editorial: Scents that Matter—from Olfactory Stimuli to Genes, Behaviors and Beyond

**DOI:** 10.3389/fnins.2016.00029

**Published:** 2016-02-09

**Authors:** Markus Fendt, Yasushi Kiyokawa, Thomas Endres

**Affiliations:** ^1^Institute for Pharmacology and Toxicology, Otto-von-Guericke University MagdeburgMagdeburg, Germany; ^2^Center of Behavioral Brain Sciences, Otto-von-Guericke University MagdeburgMagdeburg, Germany; ^3^Laboratory of Veterinary Ethology, The University of TokyoTokyo, Japan; ^4^Institute for Physiology, Otto-von-Guericke University MagdeburgMagdeburg, Germany

**Keywords:** scents, behavior, mammals, pheromones, kairomones, olfactory pathways, defense mechanisms

Mammals can recognize a large variety of scents that give information about the environment, conspecifics, and other species. The present research topic is focused on “scents that matter,” i.e., scents that indicate stimuli which are crucial for the survival of an organism. These can be positively related stimuli like the smell of familiar conspecifics, mating partners, or food, but also negatively related stimuli like the scent of potential predators, spoiled food, or territorial and aggressive conspecifics.

A prerequisite for this important role of scents in animals' lives is that they can be well detected and recognized. During the last decades, our understanding of olfactory perception has been largely improved, mainly inspired by the work of Linda Buck and Richard Axel (e.g., Buck and Axel, 1991), which was awarded by the Nobel Prize in 2004. Many of the scents studied in this research topic are processed by the vomeronasal system (e.g., Haga-Yamanaka et al.; Yu), but quite often the main olfactory system is additionally involved (e.g., Rattazzi et al.). A lot of current research addresses the questions about which molecules activate which olfactory receptors and which molecular cascades are modulated by these receptors, or how the different olfactory receptors and the two olfactory systems work together. In the current research topic the articles of Ben-Shaul, Kelliher and Munger, Rattazzi et al. and Yu provide new perspectives in this interesting field of research.

Besides the detection mechanisms of relevant scents, many studies are focusing on the behavioral changes induced by these scents. Most of these studies are analyzing scents signaling potential dangers. One reason for focusing on danger-signaling odors may be that the behavioral effects of these scents are easier to be induced and measured. In addition, it is widely believed that these scents are more critical for fostering the survival of animals. Basically, such danger-signaling scents with aversive-like effects are classified as (a) kairomones, which are emitted by another species such as predators (e.g., Apfelbach et al.; Osada et al.) or (b) pheromones, that are emitted by conspecifics such as alarm pheromones (e.g., Kobayashi et al.; Breitfeld et al.). Both classes of scents warn about a potential threat, which is intended in the case of pheromones, but unintended in the case of kairomones as they lead to a detriment of the emitter (see Nielsen et al.). It is widely believed that predator odors and alarm pheromones are innately recognized, as these stimuli are still effective in laboratory animals that have lived many generations in the absence of predators (Apfelbach et al.; Fendt et al., 2005).

In addition to the general impact of predator odors on the behavior of prey animals, an interesting line of research is the identification of active components in these scents. In the case of predator odors, several molecules have been identified so far: trimethlythiazoline (Taugher et al.; Fortes-Marco et al.; summarized in Rosen et al.), different pyrazines (Osada et al.), and pyridines (Brechbühl et al.), or 2-phenylethylamine (Ferrero et al., 2011). In the present research topic, a number of studies demonstrating that these compounds are able to induce a wide array of defensive responses in laboratory rodents such as avoidance behavior (Wernecke and Fendt; Brechbühl et al.; Fortes-Marco et al.), freezing (Taugher et al.; Fortes-Marco et al.), risk assessment behavior (Breitfeld et al.), or an inhibition of appetitive-like behavior (Kobayashi et al.), as well as physiological changes like a modulation of blood pressure (Brechbühl et al.), or breathing (Taugher et al.). Although these single molecules have the advantage that they can be better controlled in an experimental procedure (e.g., concentration), the natural scents, i.e., blends, are usually more efficient in inducing behavioral changes (summarized in Apfelbach et al.).

The neural mechanisms underlying the behavioral and physiological changes induced by danger-signaling scents are meanwhile partly understood. In the current research topic, studies are focused on brain sites like the bed nucleus of the stria terminalis (Breitfeld et al.; Taugher et al.), the medial amygdala (Carvalho et al.), the periaqueductal gray (Canteras et al.), and different subnuclei of the hypothalamus (Canteras et al.; Kobayashi et al.). Interestingly, these brain sites are of minor or no importance for learned fear whose neural basis is well understood (Fendt and Fanselow, 1999; LeDoux, 2012), suggesting a clear neuronal differentiation between innate and learned fear.

In fear learning, the danger-predicting property of a stimulus is learned by Pavlovian associative learning. Of course, olfactory stimuli can be used for such associative learning, either as unconditioned (Yuan et al.; Fortes-Marco et al.) or conditioned stimuli (Ferry et al.; Yuan et al.). The latter means that a scent without emotional valance can gain danger-predicting, i.e., fear-inducing, properties. Notably, even if a stimulus from another sensory modality is used as a conditioned stimulus in such a fear learning experiment, scents may still play some roles, since they are usually part of the experimental context (e.g., conditioning box, experimenter) and may be associated with the danger simultaneously. In fear learning, the lateral amygdala is important for associating a discrete cue with a danger stimulus, whereas the hippocampus plays an important role in contextual fear learning. Interestingly, novel work of the present research topic demonstrated that different regions of the hippocampus have different roles during contextual fear conditioning with odors (Yuan et al.). In addition to the hippocampus, several cortical areas such as the entorhinal cortex are involved in contextual fear learning (Ferry et al.).

So far, there is little research on the effects of danger signaling scents in humans. However, the defensive behaviors induced by danger-predicting scents and the respective physiological changes observed in animals are connected to anxiety in humans. Therefore, one perspective is that a deeper understanding of the neuroanatomical and neuropharmacological basis of odor-induced fear in animals may also help to find new treatment strategies for anxiety disorders in humans.

As noted above, scents can also serve as positive stimuli. This is of specific interest in the context of social behavior (Wöhr; Noack et al.; Fuzzo et al.) and foraging (Kelliher and Munger). These aspects are also covered by several articles in this special issue. It has been shown that one important function of these scents is to help to recognize social partners (Ben-Shaul; Noack et al.). Thereby, they induce and modulate a variety of behaviors, including ultrasonic calls which are typical for pleasant situations (Wöhr). In the case of social buffering, the scent of a conspecific is able to reduce fear (Fuzzo et al.). These two, quite different effects of social scents are mediated by different subnuclei of the amygdala (Fuzzo et al.; Noack et al.). Notably, there is also potential for translational research with “social scents.” For example, a genetic mouse model of autism is less able to modulate ultrasonic vocalization in response to familiar scents (Wöhr).

The present research topic nicely represents the different approaches used in “olfactory research” of relevant scents. These approaches include cell biology, genetics, behavioral pharmacology, neuroanatomy, as well as computational neuroscience. Scientists from all these fields work effectively together to unravel the mechanisms of how scents matter in humans and animals.

We are grateful to all contributors of this research topic. Eighty-five different authors from 10 different countries contributed with research and review articles. Furthermore, we thank the reviewers which helped us and the authors to create an interesting and high-quality research topic.

We hope that you enjoy reading this research topic as much as we have enjoyed editing it.

## Author contributions

MF wrote the first draft of the editorial, all authors revised the manuscript and approved the final version of it.

### Conflict of interest statement

The authors declare that the research was conducted in the absence of any commercial or financial relationships that could be construed as a potential conflict of interest.
